# Predictors for vascular cognitive impairment in stroke patients

**DOI:** 10.1186/s12883-016-0638-8

**Published:** 2016-07-26

**Authors:** Xiangliang Chen, Lihui Duan, Yunfei Han, Ling Tian, Qiliang Dai, Shang Wang, Ying Lin, Yunyun Xiong, Xinfeng Liu

**Affiliations:** 1 Department of Neurology, Jinling Hospital, Medical School of Nanjing University, 305# East Zhongshan Road, Nanjing, 210002 China; 2 Department of Neurology, Nanjing First Hospital, Nanjing Medical University, Nanjing, China; 3 Department of Neurology, Jinling Hospital, Southern Medical University, Nanjing, China; 4 Department of Cardiology, Nanjing Drum Tower Hospital, Medical School of Nanjing University, Nanjing, China

**Keywords:** Vascular cognitive impairment, Lacune, White matter hyperintensities, Stroke, Neuropsychology

## Abstract

**Background:**

Around two thirds stroke patients may suffer from vascular cognitive impairment (VCI). Our previous study has validated the NINDS-CSN harmonization standard for VCI diagnosis in Chinese. In this study, we aimed to investigate the predictors for VCI in Chinese post-stroke patients.

**Methods:**

We compared epidemiological, clinical, and neuroimaging data (number, size and location of acute infarcts and lacunes, severities of white matter hyperintensities and brain atrophy) between stroke patients with and without VCI. Univariate and logistic regression analyses were utilized to determine VCI predictors.

**Results:**

Fifty-six consecutive patients (age, 63.8 ± 8.3 years; female, 37.5 %) were recruited at a mean interval of 7.1 months after stroke onset, and 31 (55.4 %) patients were diagnosed with VCI based on a validated 60-min neuropsychological battery. VCI patients were older (*p* = 0.023), less educated (*p* = 0.001), more likely to be female (*p* < 0.001), had a recurrent stroke (*p* = 0.028), and described higher apathy (*p* = 0.022) and worse pre-stroke cognition (*p* = 0.048) than cognitively normal patients. Lower educational level (adjusted odds ratio [OR] 0.750, 95 % confidence interval [CI], 0.573–0.981; *p* = 0.035), female sex (adjusted OR 8.288, 95 % CI, 1.522–45.113; *p* = 0.014), recurrent stroke (adjusted OR 11.327, 95 % CI, 1.335–96.130, *p* = 0.026), and global cortical atrophy (adjusted OR 5.730, 95 % CI, 1.128–29.101, *p* = 0.035) were independently associated with VCI in post-stroke patients.

**Conclusions:**

Lower education, female sex, recurrent stroke and global cortical atrophy were associated with VCI in Chinese stroke patients.

**Electronic supplementary material:**

The online version of this article (doi:10.1186/s12883-016-0638-8) contains supplementary material, which is available to authorized users.

## Background

Stroke survivors are prone to develop cognitive impairment, with a frequency of 62.6 % at 3 months post-stroke [1], and persisted long-term functional effects [2]. This prolonged cognitive sequela of stroke has been widely used to study vascular cognitive impairment (VCI). However, most studies have either focused on dementia [3], or used diverse diagnostic criteria with various cognitive scales [4]. In 2006, the National Institute of Neurological Disorders and Stroke and Canadian Stroke Network (NINDS-CSN) established common standards for VCI assessments [5], and our previous work validated the Chinese adaption of neuropsychological scales in mild stroke patients [6].

VCI may result directly from an acute stroke or from underlying chronic brain lesions, such as white matter hyperintensities (WMHs) and subclinical lacunes [7]. However, the predictors for VCI in post-stroke patients was equivocal [3, 8, 9]. In some studies, WMHs [3, 8], lacunes [10, 11], silent brain infarctions [12], and brain atrophy [3, 10] had been associated with VCI after stroke. However, others reported that large infarcts were associated with greater likelihood of cognitive dysfunction, but found no significant effect of WMHs, silent infarcts or cerebral atrophy in predicting post-stroke VCI [13], or had not found any significant association after multivariable adjustment [12, 14].

Although VCI used to be diagnosed only in the context of a cerebral infarction, recent studies have highlighted the importance of pre-existing brain lesions as a key determinant for incident VCI in post-stroke patients [3, 8]. Moreover, in the investigation of factors related to VCI development, few studies have applied a population-validated NINDS-CSN neuropsychological protocol to VCI diagnosis [1]. To investigate the predictors for post-stroke VCI, we collected epidemiological, clinical and neuroimaging data in detail. We hypothesized that chronic brain lesions (e.g., old infarcts, WMHs, and brain atrophy) would be significant predictors of the development of VCI after stroke.

## Methods

### Participants

Participants were consecutive patients with an MRI scan for an acute ischemic stroke, aged 50 years or older, admitted to a teaching hospital from January to June 2013. Additional inclusion criteria for neuropsychological assessment were an available informant who was knowledgeable with the patient’s cognitive performance on a daily or next to daily basis for at least 10 years prior to recruitment, informed consent, and absence of severe motor and language disabilities hindering cognitive evaluation. Exclusion criteria for this study were a history of hemorrhagic stroke, traumatic brain injury, Parkinson disease, psychiatric disorders known to influence cognitive function, or a pre-existing cognitive impairment according to the Informant Questionnaire on Cognitive Decline in the Elderly (IQCODE), a screening instrument validated in Chinese, scoring 3.4 or more [15]. The institutional review board at Jinling Hospital approved the study, and all participants provided written informed consent.

### Neuropsychological assessment

Patients’ cognitive and psychological status was assessed at 3 to 12 months after stroke, using the 60-min Chinese version of NINDS-CSN neuropsychological protocol. The protocol examined the following four cognitive domains: executive/activation (animal naming [16], WAIS-III Digit symbol-coding [17], trail making test, parts A and B [18]), language (modified Boston naming test [19]), visuoconstruction (Rey-Osterrieth Complex Figure Test [RCFT]-copy trial [20]), and memory (delayed recall on the revised Hopkins verbal learning test [21] and RCFT [20]). Besides, the Chinese version of Mini-Mental State Examination (MMSE) and the Montreal Cognitive Assessment (MoCA), Beijing version were evaluated to test global cognitive function; the Geriatric Depression Scale (GDS) and the Apathy Evaluation Scale (AES) were used to assess depressive and apathetic symptoms, respectively; the Neuropsychiatric Inventory Questionnaire (NIQ) was administered to probe behavioral domains; and the IQCODE was also completed by the informant to obtain the pre-morbid history of cognitive status. All neuropsychological tests were administered in each subject’s primary language of Mandarin Chinese. For each domain, cognitive impairment was identified if more than half of the tests had a score >1.5 SD below age and education matched means of controls [6]. For example, the executive/activation domain consisted of 4 tests, and was classified as having an abnormal function if patients scored >1.5 SD below the means on at least 2 tests. The subject was categorized as a VCI patient when cognitive impairment was observed in at least one domain, and in the absence of pre-stroke cognitive impairment.

### Medical history

We retrospectively collected data on demographics and clinical factors regarding the index event from medical records. Basic information included age, sex, years of education, and body mass index. Vascular risk factors that we recorded included hypertension (defined as presenting with a history of hypertension, or under antihypertensive treatment), diabetes mellitus (defined by fasting plasma glucose ≥7.0 mmol/L, or 2-h postprandial glucose ≥11.1 mmol/L, or the use of insulin/oral hypoglycemic medication), hyperlipidemia (defined as total cholesterol ≥5.2 mmol/L, or low-density lipoprotein cholesterol ≥2.6 mmol/L, or triglyceride ≥1.70 mmol/L, or being treated with lipid-lowering drugs), atrial fibrillation (diagnosed according to electrocardiogram), ischemic heart disease (defined as a history of myocardial infarction or angina pectoris), recurrent stroke, smoking, and current alcohol use (drinking >2 units per day). In addition, stroke subtype (Trial of Org 10172 in Acute Stroke Treatment, TOAST criteria) and severity (National Institutes of Health Stroke Scale, NIHSS score) of stroke were also recorded.

### Neuroimaging examination

Routine non-contrast brain computed tomography was initially performed. All patients underwent brain MRI on a 1.5-T or 3.0-T scanner (GE Healthcare, Milwaukee, WI, USA), including sequences of axial T1-weighted imaging (TR/TE = 350/2.5 ms), axial T2-weighted imaging (TR/TE = 4000/98 ms), diffusion-weighted imaging (DWI) (TR/TE = 3000/91 ms), and coronal T2 fluid-attenuated inversion recovery (FLAIR) (TR/TE = 8000/93 ms), with slice thickness 5 mm, spacing between slices 6.5 mm, field of view 90.625 mm for T1/T2-weighted imaging, 100 mm for DWI and 81.25 mm for FLAIR, and matrix of 320 × 260, 512 × 416, 256 × 256 and 512 × 464 for T1, T2-weighted imaging, DWI and FLAIR, accordingly. All MRI images were read by X. C. who was blinded to subjects’ clinical or neuropsychological information.

The assessment of acute and chronic brain lesions were based on MRI. An acute infarction was defined as a lesion that showed DWI hyperintensity with corresponding apparent diffusion coefficient hypointensity [22]. Acute ischemic characteristics with regard to infarct number, size and location were collected. Lesion size was recorded in maximum axial diameter by using the built-in software Numaris/4 (syngo MR B17; Siemens). Lesion locations were classified as frontal lobe, parieto-occipital lobe, temporal lobe, basal ganglia, thalamus or the infratentorial area. WMHs were visually rated on T2-weighted and FLAIR images using the age-related white matter change (ARWMC) scale [23]. Large and small old infarcts evident on MRI were recorded. Small old infarcts, termed as lacunes, were CSF-like lesions of between 3 mm and 15 mm in diameter, with a surrounding rim of hyperintensity on FLAIR and T1-weighted images [24]. Brain atrophy was estimated by a 4-point rating scale for the assessment of global cortical atrophy (GCA) [25], and a 5-point rating scale for medial temporal lobe atrophy (MTA) [26], with GCA being defined as 1 point or more and MTA ≥2 points. The presence of cerebral artery stenosis was determined by magnetic resonance angiography, computed tomography angiography, or digital subtraction angiography.

To test intra-rater agreement of the neuroimaging evaluation, 10 randomly selected scans were re-read by the same rater (X. C.), blinded to previous rating scores. The intra-rater agreement was then assessed using intraclass correlation coefficients (ICC).

### Statistical analysis

Descriptive statistics were calculated for demographics, clinical, and neuroimaging variables in patients with and without VCI. Group comparisons of categorical variables were performed with the *χ*2 test, and comparisons of continuous variables were done by the independent sample *t* test, or Mann–Whitney *U* test in case of skewed distributions. Effect sizes were estimated in a common form of Cohen’s *d*, and the intervals for Cohen’s *d* were: 0–0.1, no effect; 0.2–0.4, small effect; 0.5–0.7, intermediate effect; 0.8 and higher: strong effect [27]. Variables that were important risk factors for VCI based on prior knowledge, except those highly correlated, were introduced as independent variables in multivariate stepwise logistic regression analysis. *p* < 0.05 indicated statistical significance. All analyses were performed using the SPSS Statistics for Windows, version 17.0.

## Results

Between January and June 2013, 165 stroke patients with MRI scans were screened, of whom 56 patients underwent neuropsychological assessments (mean age, 63.8 ± 8.3 years, 37.5 % were women). Median NIHSS score on admission was 3 (interquartile range [IQR], 1–5.75). The interval between stroke onset and cognitive assessment was 7.1 months (SD = 2.3), while the interval between MRI examination and cognitive assessment was 6.9 months (SD = 2.3). Comparisons were made between patients with (*n* = 31, 55.4 %) and without (*n* = 25, 44.6 %) VCI.

Table 1 compared the demographics, risk factors, and stroke features between the two groups. VCI patients were older, less educated, more likely to be female, and more likely to have a recurrent stroke than cognitively normal patients. No between-group differences were observed in terms of stroke subtype, stroke severity and other vascular risk factors. The onset-to-test time was similar between groups.

**Table 1:** Clinical characteristics comparing patients with and without VCI after stroke

Characteristics	Patients without VCI (*n* = 25)	Patients with VCI (*n* = 31)	*p* value	Cohen’s *d*
Demography				
Age (years)^a^	61.0 ± 6.9	66.0 ± 8.8	0.023	0.63
Sex (female)	3 (12.0 %)	18 (58.1 %)	<0.001	1.07
Education (years)^a^	11.2 ± 3.7	7.5 ± 4.3	0.001	0.92
Body mass index (kg/m^2^)^a^	24.8 ± 2.3	25.6 ± 4.0	0.416	0.25
Vascular risk factors				
Hypertension^b^	19 (76.0 %)	27 (87.1 %)	0.281	0.29
Diabetes mellitus^b^	10 (40.0 %)	13 (41.9 %)	0.884	0.04
Hyperlipidemia^b^	19 (76.0 %)	22 (71.0 %)	0.672	0.11
Atrial fibrillation^b^	1 (4.0 %)	1 (3.2 %)	0.698	0.04
Ischemic heart disease^b^	4 (16.0 %)	2 (6.5 %)	0.237	0.31
Current/former smoker^b^	16 (64.0 %)	13 (41.9 %)	0.100	0.45
Alcohol abuse^b^	7 (28.0 %)	3 (9.7 %)	0.075	0.49
Recurrent stroke^b^	2 (8.0 %)	10 (32.3 %)	0.028	0.62
Stroke subtype by TOAST			0.592	0.38
Large-artery atherosclerosis^b^	10 (40.0 %)	17 (54.8 %)		
Small-artery occlusion^b^	10 (40.0 %)	11 (35.5 %)		
Cardioembolism^b^	1 (4.0 %)	1 (3.2 %)		
Undetermined cause^b^	4 (16.0 %)	2 (6.5 %)		
Stroke severity				
NIHSS score^a^	3.2 ± 3.2	4.4 ± 3.7	0.236	0.35
Onset-to-test time (months)^a^	6.7 ± 2.3	7.4 ± 2.4	0.294	0.30

*TOAST* trial of org 10172 in acute stroke treatment, *NIHSS* National Institute of Health stroke scale

^a^mean ± SD, independent sample *t* test

^b^number (%), *χ*2 test

Comparisons of neuropsychological assessments between stroke patients with and without VCI were presented in Table 2. Patients with VCI scored worse than those without VCI on individual tests. Global cognitive functioning, as assessed by MMSE and MoCA, was also significantly worse in patients with VCI. Emotional tests indicated that VCI patients described higher apathy than cognitively normal patients. Pre-stroke cognitive status as measured by the IQCODE was also worse for the VCI group (*p* = 0.048, cohen’s *d* = 0.55). The patients with cognitive impairment were impaired on the executive/activation domain at a percentage of 67.7 %, on the language domain of 45.2 %, on the visuoconstruction domain of 64.5 %, and on the memory domain of 77.4 %; besides, 12.9 % of the patients were impaired on a single domain. By the MMSE cutoff points for cognitive impairment (17/18 for illiterates, 19/20 for individuals with 1 to 6 years of education, and 24/25 for those with 7 or more), only 19.4 % of the patients were below the cutoff.

**Table 2:** Comparison of neuropsychological assessments between patients with and without VCI after stroke

Neuropsychological tests	Patients without VCI (*n* = 26)	Patients with VCI (*n* = 31)	*p* value	Cohen’s *d*
Executive/Activation				
ANT^a^	16.0 ± 3.8	11.6 ± 4.2	<0.001	1.10
WAIS-III Digit symbol-coding^a^	20.6 ± 4.5	10.8 ± 5.8	<0.001	1.86
TMT A time (sec)^a^	43.4 ± 9.8	111.5 ± 74.2	<0.001	1.29
TMT B time (sec)^a^	104.5 ± 31.0	200.7 ± 78.4	<0.001	1.62
Language				
Modified BNT^a^	11.4 ± 2.1	8.2 ± 2.4	<0.001	1.42
Visuospatial				
RCFT copy^a^	34.7 ± 1.2	28.1 ± 7.0	<0.001	1.31
Memory				
HVLT-R delayed recall^a^	7.0 ± 2.3	3.3 ± 2.1	<0.001	1.70
RCFT delayed recall^a^	19.0 ± 4.7	9.8 ± 7.3	<0.001	1.50
Supplemental tests				
MMSE score^a^	28.1 ± 1.3	24.6 ± 3.2	<0.001	1.43
MoCA score^a^	22.7 ± 3.2	16.4 ± 4.4	<0.001	1.64
GDS score^a^	2.7 ± 1.9	3.9 ± 4.1	0.146	0.38
AES score^a^	59.4 ± 5.7	54.4 ± 9.3	0.022	0.65
NIQ score^b^	9 (0.5–16.0)	4 (0–20.0)	0.714	0.10
IQCODE^a^	3 (3.0–3.1)	3.1 (3.0–3.2)	0.048	0.55

*ANT* Animal naming test, *TMT* trail making test, *BNT* Boston naming test, *RCFT* rey-osterrieth complex figure test, *HVLT-R* revised Hopkins verbal learning test, *MMSE* mini-mental state examination *MoCA* montreal cognitive assessment, *GDS* geriatric depression scale, *AES* apathy evaluation scale, *NIQ* neuropsychiatric inventory questionnaire

^a^mean ± SD, independent sample *t* test

^b^median (IQR), Mann–Whitney *U* test

The neuroimaging features in each group were displayed in Table 3. The reproducibility after a mean duration of 34.5 days (SD = 5.8) showed excellent intra-rater agreement in rating WMHs by ARWMC total score (ICC, 0.871; 95 % confidence interval [CI], 0.482–0.968), and MTA (ICC, 0.947; 95 % CI, 0.786–0.987) and GCA(ICC, 0.957; 95 % CI, 0.827–0.989). By group comparisons, a trend was noted in VCI patients with regard to larger acute lesions, more old infarcts, larger lacunar sizes and greater GCA (cohen’s *d* ranged from 0.42 to 0.48). However, ARWMC total score, MTA, and locations of acute and chronic lesions (effect sizes ranged from 0.02 to 0.40) showed no differences between groups (Additional file 1).

**Table 3:** Comparison of neuroimaging features between patients with and without VCI after stroke

Neuroimaging features	Patients without VCI (*n* = 25)	Patients with VCI (*n* = 31)	*p* value	Cohen’s *d*
Patients with acute lesion^a^	24 (96.0 %)	31 (100.0 %)	0.446	0.30
No. of acute lesions^b^	1 (1–2.0)	1 (1–3.0)	0.184	0.36
Lesion size (mm)^c^	19.0 ± 11.3	26.0 ± 20.5	0.112	0.42
Chronic brain changes				
Patients with large old infarct^a^	1 (4.0 %)	6 (19.4 %)	0.091	0.47
Patients with lacunes^a^	18 (72.0 %)	25 (80.6 %)	0.446	0.20
No. of lacunes^c^	3.0 ± 3.3	4.4 ± 4.2	0.180	0.37
Lacunar size (mm)^c^	5.7 ± 4.3	7.6 ± 4.48	0.107	0.44
ARWMC total score^c^	5.6 ± 3.8	6.6 ± 4.0	0.338	0.26
Presence of MTA^a,d^	5 (20.0 %)	4 (12.9 %)	0.360	0.19
Presence of GCA^a,e^	7 (28.0 %)	15 (48.4 %)	0.120	0.48
Artery stenosis			0.247	0.56
No stenosis^a^	6 (24.0 %)	4 (12.9 %)		
Intracranial stenosis^a^	12 (48.0 %)	20 (64.5 %)		
Extracranial stenosis^a^	7 (28.0 %)	5 (16.1 %)		
Tandem stenosis^a,f^	0	2 (6.5 %)		
Stenosis severity			0.666	0.12
None^a^	6 (24.0 %)	4 (12.9 %)		
Mild stenosis (0–50 %)^a^	7 (28.0 %)	7 (22.6 %)		
Moderate stenosis (50 %–70 %)^a^	2 (8.0 %)	6 (19.4 %)		
Severe stenosis (70–99 %)^a^	2 (8.0 %)	9 (29.0 %)		
Occlusion^a^	8 (32.0 %)	5 (16.1 %)		

*ARWMC* age-related white matter change scale, *MTA* medial temporal lobe atrophy, *GCA* global cortical atrophy

^a^number (%), *χ*2 test

^b^median (IQR), Mann–Whitney *U* test

^c^mean ± SD, independent sample *t* test

^d^Presence of MTA was defined as a rating of 2 points or more

^e^Presence of GCA was defined as a rating of 1 point or more

^f^Tandem stenosis was defined as more than one stenotic lesion with severity ≥50 % in the same vascular distribution

To explore the independent association of acute or chronic brain lesions with VCI, we chose important variables based on prior knowledge, including age, sex, education, hypertension, diabetes, recurrent stroke, lacunar number/lacunar size, ARWMC score, presence of MTA, GCA, AES and GDS score, into a multivariate logistic regression model (Table 4). Lower educational level (odds ratio [OR] 0.750, 95 % confidence interval [CI], 0.573–0.981; *p* = 0.035), female sex (OR 8.288, 95 % CI, 1.522–45.113; *p* = 0.014), a recurrent stroke (OR 11.327, 95 % CI, 1.335–96.130, *p* = 0.026), and global cortical atrophy (OR 5.730, 95 % CI, 1.128–29.101, *p* = 0.035) were identified as independent predictors for post-stroke VCI.

**Table 4:** Risk factors associated with VCI in multivariate logistic regression

Risk factors	Odds ratio	95 % Confidence interval	*p* value
Education	0.750	0.573–0.981	0.035
Female sex	8.288	1.522–45.113	0.014
Recurrent stroke	11.327	1.335–96.130	0.026
Presence of GCA	5.730	1.128–29.101	0.035

Model adjusted for age, hypertension, diabetes, lacunar number/lacunar size, age-related white matter change score, medial temporal lobe atrophy, apathy and depression scores

*GCA* global cortical atrophy

## Discussion

This study suggested that risk factors for VCI in post-stroke patients included lower education, female sex, recurrent stroke and global cortical atrophy.

Stroke serves as an important factor in VCI. In our study, 55.4 % of the stroke patients suffered VCI, which was lower than that (62.6 %) of a recent study in Korea [1]. However, participants in our study had a longer interval between neuropsychological tests and stroke (7.1 months vs. 3 months), which could possibly allow stroke recovery. Evaluations took place 3 to 12 months after stroke onset. Therefore, some participants were further along in their recovery than others. While onset-to-test time did not vary between patients with and without VCI, a long time span may have reduced the sensitivity of the neuropsychological tests.

Recurrent stroke was an independent risk factor for VCI in our study, paralleling previous findings that recurrent stroke would at least double the rates of dementia than first-ever stroke [28]. The underlying mechanism may be that an increased volume of brain injury would reduce the threshold for manifestation of subclinical neurodegenerative disorders. Compared with cognitively normal patients, VCI patients tended to show a larger infarct size with a medium effect size of the differences. Earlier studies had generally shown an inconsistent relationship between size and number of infarcts and cognitive impairment [3, 13], this could possibly be explained by the uncertainty of cortical involvement, and differences in distribution of hypoperfusion [7]. Though cortical involvement was considered to be associated with more impaired cognitive function [13], the scientific evidence for it was rare and conflicting. One recent studies showed a comparable performance between cortical and subcortical infarctions [29], and our study did not show between-group differences regarding lesion locations as well. Nevertheless, these findings were based on a cross-sectional design, and how the change of an acute lesion over time could affect cognitive progression could be further explored.

The role of chronic brain lesions on the development of post-stroke VCI was substantial [7]. Patients with lacunes have been reported to have poorer performance in multiple cognitive domains and steeper decline in cognitive function in different cohorts, such as the Cardiovascular Health Study, the Rotterdam Study and the Epidemiology of Dementia in Singapore study [10–12]. Our study likewise indicated a trend of association between lacunar size and VCI. The possible explanation was that an increase in lacunar volume was associated with decline in executive function [30], besides, it was demonstrated that the silent lacunar infarct was related to cortical atrophy, which may also impair cognition [10]. Recent surveys underlined the importance of WMHs, MTA as independent risk factors for VCI after stroke [3, 8], but in this study, VCI patients did not show an association with WMHs or MTA. This might be due to the exclusion of patients with pre-stroke cognitive impairment in our study; for individuals with more severe WMHs and more atrophy are prone to display a worsening of cognitive ability, which could account for their apparent associations with post-stroke VCI [7]. Severe WMHs were mainly associated with frontal lobe dysfunction, which was considered to be a consequence of damaged neural transmission and interneural connection, however, novel evidence implicated that the effects of WMHs on cognitive performance might be mediated by cortical thickness [31]. Therefore, in some reports, more extensive WMHs were shown to be related to cognitive impairment, but the association did not persist in multivariate modeling [12, 14]. Another study from Finland also suggested a loss of significance for the correlation between MTA and post-stroke dementia after excluding individuals with pre-stroke dementia [32].

In this study, female sex and education were independently associated with VCI in post-stroke patients, concurring with previous findings [33, 34]. Female sex may play a role in poststroke cognitive impairment by related degenerative pathology, which would interact with vascular pathology as well [35], and unsurprisingly, greater prestroke cognitive decline was observed in VCI patients in our study. The beneficial effect of education could be that a higher education level indicated better cognitive reserve, in this way, patients with higher reserve would show an altered, compensatory network to maintain function in the face of age-related pathophysiological changes [36].

Various definitions of VCI would hinder its identification. Based on the common standards proposed by the NINDS-CSN, our study may be one of the first studies using population validated criteria for VCI diagnosis [1, 5]. Besides, this study will in part contribute to a future meta-analysis on the subject whether patients with cognitive impairment were associated with direct infarct lesions, or the underlying chronic brain lesions.

Limitations of the study were as follows. First, this was a single-center study, the sample size was small and was heterogeneous with regard to age, stroke subtype, and cognitive status, limiting the statistical power, and increasing the possibility of type II errors. Thus, null findings with meaningful effect sizes such as larger size of acute infarct and lacunes, and cortical atrophy, may be negatively affected due to low statistical power. Second, the study sample was hospital-based and most patients suffered a relatively mild stroke, so our results may not generalize to those with more severe or very mild stroke, or to those who show specific cognitive deficits (e.g., aphasia) that precluded proper cognitive assessment. Third, pre-stroke cognitive status was merely measured by IQCODE due to the cross-sectional nature, hence patients with subtle cognitive impairment may be included. Fourth, both 1.5 T and 3.0 T MRIs were used, which could introduce magnetic bias for their sensitivity to lesions may be different. Fifth, we measured MTA, GCA and WMHs using visual rating scale, and lacunes by size and number rather than quantitative calculation, further larger, multi-center studies that use automated unbiased measurements are needed to confirm the observations in the present study.

## Conclusion

In conclusion, lower education, female sex and recurrent stroke were predictors for VCI in Chinese post-stroke patients. Chronic brain lesions such as lacunes and cortical atrophy might be potential imaging biomarkers of VCI, which could be examined in future studies. In addition, effort should be made to optimize secondary stroke prevention and to increase cognitive reserve, for the purpose of delaying the clinical expression of VCI. Further preclinical studies are warranted to investigate the underlying pathogenesis as well as targeted interventions in long term VCI prevention and treatment.

## Abbreviations

AES, apathy evaluation scale; ARWMC, age-related white matter change; FLAIR, fluid-attenuated inversion recovery; GCA, global cortical atrophy; GDS, Geriatric depression scale; ICC, intraclass correlation coefficients; IQCODE, the informant questionnaire on cognitive decline in the elderly; MMSE, mini-mental state examination; MoCA, montreal cognitive assessment; MTA, medial temporal lobe atrophy; NINDS-CSN, National Institute of Neurological Disorders and Stroke and Canadian Stroke Network; NIQ, neuropsychiatric inventory questionnaire; OR, odds ratio; RCFT, Rey-Osterrieth complex figure test; TOAST, trial of org 10172 in acute stroke treatment; VCI, vascular cognitive impairment; WMH, white matter hyperintensities

## Additional file

Additional file 1: Comparison of lesion locations between patients with and without VCI after stroke. Data showed no between-group differences concerning the locations of acute and chronic brain lesions (effect sizes ranged from 0.02 to 0.40). (DOCX 15 kb)
