# Supplementary material for: Predictors for vascular cognitive impairment in stroke patients
Source: BMC Neurol. 2016 Jul 26;16:115. doi: 10.1186/s12883-016-0638-8 (PMC4962370; doi:10.1186/s12883-016-0638-8)
Supplement: Additional file 1: — Comparison of lesion locations between patients with and without VCI after stroke. Data showed no between-group differences concerning the locations of acute and chronic brain lesions (effect sizes ranged from 0.02 to 0.40). (DOCX 15 kb) [file 12883_2016_638_MOESM1_ESM.docx]

| Supplemental File 1 Comparison of lesion locations between patients with and without VCI  after stroke | | | | | |  |
| --- | --- | --- | --- | --- | --- | --- |
| Lesions and locations | Patients without VCI (n=25) | Patients with VCI (n=31) | *p* value | Cohen's *d* |  |  |
| Location of acute ischemic lesions | | | | |  |  |
| Basal ganglia | 6 (23.1%) | 6 (20.0%) | 0.674 | 0.11 |  |  |
| Thalamus | 3 (11.5%) | 3 (10.0%) | 1.000 | 0.07 |  |  |
| Frontal lobe | 6 (23.1%) | 9 (30.0%) | 0.672 | 0.11 |  |  |
| Temporal lobe | 2 (11.5%) | 5 (13.3%) | 0.443 | 0.25 |  |  |
| Parietal-occipital lobe | 6 (26.9%) | 13 (40.0%) | 0.159 | 0.38 |  |  |
| Infratentorial region | 7 (26.9%) | 9 (30.0%) | 0.932 | 0.02 |  |  |
| Location of large old infarcts | | | | |  |  |
| Basal ganglia | 1 (3.8%) | 2 (6.7%) | 1.000 | 0.11 |  |  |
| Thalamus | 0 | 0 |  |  |  |  |
| Frontal lobe | 0 | 1 (3.3%) | 1.000 | 0.24 |  |  |
| Temporal lobe | 0 | 0 |  |  |  |  |
| Parietal-occipital lobe | 0 | 2 (6.7%) | 0.497 | 0.35 |  |  |
| Infratentorial region | 0 | 2 (6.7%) | 0.497 | 0.35 |  |  |
| Location of lacunes |  |  |  |  |  |  |
| Basal ganglia | 13 (50.0%) | 22 (73.3%) | 0.145 | 0.40 |  |  |
| Thalamus | 9 (38.5%) | 14 (43.3%) | 0.488 | 0.19 |  |  |
| Frontal lobe | 10 (34.6%) | 17 (60.0%) | 0.269 | 0.30 |  |  |
| Temporal lobe | 3 (11.5%) | 1 (3.3%) | 0.314 | 0.34 |  |  |
| Parietal-occipital lobe | 1 (3.8%) | 5 (16.7%) | 0.210 | 0.40 |  |  |
| Infratentorial region | 2 (7.7%) | 4 (13.3%) | 0.682 | 0.16 |  |  |
